# Trauma and post-traumatic stress disorder among psychiatric inpatient children and adolescents

**DOI:** 10.1080/20008198.2017.1351161

**Published:** 2017-09-29

**Authors:** Maria Belivanaki, Stavroula Ropi, Niki Kanari, John Tsiantis, Gerasimos Kolaitis

**Affiliations:** ^a^ Department of Child Psychiatry, Medical School, National and Kapodistrian University of Athens, Aghia Sophia Children’s Hospital, Athens, Greece

**Keywords:** Post-traumatic stress disorder (PTSD), sexual abuse, physical abuse, psychiatric inpatient therapy, children and adolescents

## Abstract

Posttraumatic stress disorder (PTSD) is common in young people in need of inpatient and outpatient mental health services but PTSD is underdiagnosed in clinical settings (Havens et al., 2012). Despite the high prevalence and clinical significance of early recognition of trauma exposure and PTSD in mental health settings, currently there are few empirical data that shed light on the treatment implications in acute care: 40–50% of children and adolescents up to the age of 18 have been exposed to traumatic events and 6% suffer from PTSD. In psychiatric inpatients, the percentages are 46–96% and 21–29% respectively (Gudiño et al., 2014). Young people are also at risk of presenting with psychiatric symptoms, including anxiety, depression, delinquent behaviour, separation anxiety and self-harming behaviours (Havens et al., 2012). The under-identification of PTSD symptoms in clinical settings may hinder effective treatments.

In order to examine the rates of trauma exposure and PTSD in inpatient psychiatric settings, we conducted a study of 56 children and adolescents who were admitted to the inpatient unit of the Department of Child Psychiatry, Athens University Medical School at the Aghia Sophia Children’s General Hospital, Athens, Greece, over a three-year period, Results revealed that 75% of the hospitalized children and adolescents had been exposed to violent events, either in their family or in the community. Of them, 26% (19% of the total inpatient population) had developed PTSD while the majority had presented symptoms of PTSD.

In addition, of those children and adolescents with PTSD symptoms, 60% required longer hospitalizations and need acute care treatment. Youth diagnosed with PTSD presented with high levels of clinical severity and complexity. High rates of comorbidity were also observed among those with PTSD; the main comorbid disorders found were mood disorders (75% of all cases), but also somatization, conduct, generalized anxiety and, in some, eating and psychotic disorders. In 73% of PTSD cases and in 48.4% of those exposed to traumatic events, suicidal attempts were reported (67 and 53% had reported suicidal ideation) while the percentage of the inpatients with attempted suicides as a result of other psychiatric disorders was 19%. Youth with exposure to trauma and PTSD presented at admission with major impairment in functioning in several areas as measured by the Children’s Global Assessment Scale (C-GAS) (mean score = 32.8, with 91–100 showing superior functioning in all areas). Nevertheless, there is limited research on functioning (i.e. academic performance, peer relationships) in youth with PTSD which is much needed considering that it affects executive cognitive functions and mood. It is worth reporting that in our study, the systematic use of Kiddie-Schedule for Affective Disorders and Schizophrenia-Present and Lifetime version (K-SADS-PL) in the inpatient unit mainly led to the identification of trauma and consequent PTSD. The discrepancy between rates of PTSD at the intake and later through standardized assessment was large as also reported in other studies (e.g. Havens et al., 2012).

Despite the high prevalence of PTSD, many studies, including our study, show that PTSD is underdiagnosed in psychiatric acute care settings. An adequate assessment (Verlinden et al., 2015) is critical and ensures the best available level of care and effective treatment. With 70% of youth experiencing exposure to environmental traumatic stressors, the above study stresses the significance of early screening of trauma-related events in psychiatric patients. The identification through systematic questioning with structured or semi-structured interviews of youth’s exposure to trauma should be followed by trauma related therapies inside the inpatient setting and/or on an outpatient basis. We also propose that future research should focus on the clinical and social needs of the trauma exposed youth and their societal background and the prevention of manifestations related to psychiatric disorders.
